# Acute Pancreatitis After the Use of Belimumab in a Patient With Systemic Lupus Erythematosus: Case Report and Review of Literature

**DOI:** 10.7759/cureus.22540

**Published:** 2022-02-23

**Authors:** Izza Bazigh, Mohamad Asfour, Salman Muddassir, Sami Mughni

**Affiliations:** 1 Internal Medicine, Oak Hill Hospital, Spring Hill, USA; 2 Rheumatology, Oak Hill Hospital, Spring Hill, USA

**Keywords:** b-cell inhibition, drug-induced pancreatitis, severe acute pancreatitis, systemic lupus erythematosis, belimumab

## Abstract

Belimumab is a B-cell depletion therapy that has emerged as an effective and safe treatment option for Systemic Lupus Erythematosus (SLE), but ongoing phase IV trials continue to report its common and rare adverse effects. Our case report seeks to add data to the existing literature on the safety profile of belimumab.

We report an interesting and complicated case of a 30-year-old female with a 12-year history of SLE and multiple treatment failures who developed acute pancreatitis in the context of the initiation of belimumab. The temporal link between the two events made us undertake a review of literature on the efficacy and safety of belimumab. We used PubMed and Medline to shortlist eight studies that included phase III parent and extension clinical trials of belimumab that had been conducted in the past 10 years and illustrated the results in a tabulated form.

The United States Food and Drug Administration has approved belimumab as a safe and effective treatment option for SLE. The BLISS-52, BLISS-76, and BLISS-SC trials along with their extension trials showed that SRI (SLE Responder Index) was higher in the patient cohorts that were treated with IV (intravenous) or SC (subcutaneous) belimumab. According to the website “eHealthMe.com”, which tracks the incidence of adverse events from drugs by allowing people to report events, 14100 people reported side effects when taking belimumab and among them, 29 people (0.21%) reported acute pancreatitis. Time on belimumab when patients had acute pancreatitis was 1-2 years for 52% of the patients and 1-6 months for 40% of the patients; 96% of the patients were females. The age group at which it was most reported was 40-49 years.

Additional data is needed to enable a better characterization of the pathophysiology and nature of acute pancreatitis as a possible side effect of belimumab.

## Introduction

Around 300,000 to 4 million people in the United States have systemic lupus erythematosus (SLE) [1]. Treatment options for SLE are not limited to antimalarial, corticosteroids, cytotoxics and immunosuppressant drugs. Most of these drugs can cause serious adverse effects when used for the long term and some patients develop flares despite optimal treatment [2]. For decades now, newer therapeutic options have been studied for SLE but no drug received FDA (United States Food and Drug Administration) approval for over 40 years until the past decade. Belimumab received FDA approval for use in SLE in 2011. Belimumab is a B-cell depletion therapy which when used with standard of care (SoC) has emerged as a novel and effective treatment option for SLE [3]. However, post-marketing surveys continue to report data on its common and rare adverse effects. We present the case of a 30-year-old female with a 12-year-long history of SLE that had been very challenging to manage with multiple failed immunosuppressive therapies who developed acute pancreatitis in the context of the initiation of belimumab. 

## Case presentation

Our patient is a 30-year-old caucasian female who initially presented to a community rheumatology clinic 12 years ago with polyarthralgia and skin rash. The patient was found to have rheumatoid arthritis (RA) with overlapping features of SLE with mucocutaneous manifestations. She had positive antinuclear antibody (ANA), double-stranded DNA and low complement levels. Over the next 12 years, the patient failed multiple immunosuppressive therapies for different reasons. With methotrexate, patient developed transaminitis. Due to immunosuppression from mycophenolate, she was believed to have developed sigmoid diverticulitis leading to bowel perforation and colon resection. With hydroxychloroquine, the patient developed intermittent drug-induced leukocytoclastic vasculitis. Ultimately and after a significant amount of time spent on education, counseling and reviewing of old medical records, the patient was started on belimumab infusion. The patient’s BMI at the time was 37.

**Figure 1:**
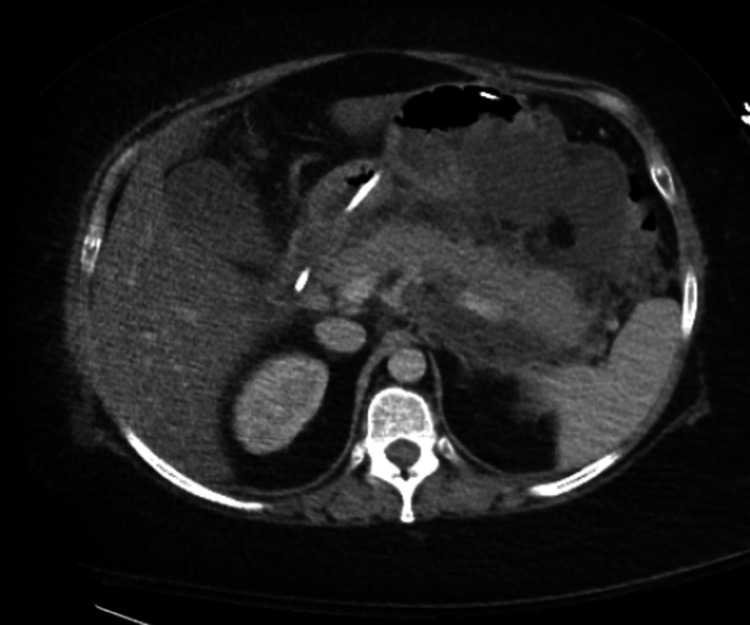
CT evidence of acute pancreatitis with peri-pancreatic necrosis.

After four months of monthly belimumab infusions, she was switched to weekly subcutaneous (SC) belimumab due to lack of response. Three months after switching to SC belimumab, the patient was admitted for epigastric pain. Computerized Tomography (CT) of the abdomen and pelvis showed evidence of acute pancreatitis. The lipase level was found to be elevated at 969 U/L. After supportive care, the patient was discharged. Two weeks later, the patient presented with the same symptoms. CT abdomen and pelvis showed pancreatitis with peripancreatic stranding in fluid and necrosis (Figure 1). The lipase level was elevated to 11427 U/L. Rheumatology service was consulted for evaluation of possible autoimmune etiology of pancreatitis.

The patient was worked up for autoimmune pancreatitis (AIP). Due to the isolated involvement of the pancreas and the fact that her IgG 4 level was 1 mg/dL (normal: 2-96 mg/dL), she was believed to have type 2 AIP. The patient's CT abdomen and pelvis showed persistent diffuse edema of the pancreas with peripancreatic stranding of fat and fluid but no characteristic sausage shaped appearance of AIP. Other considerations included gastrointestinal manifestation of SLE and drug-induced pancreatitis. Irrespective of the etiology, she was started on steroids. Contrary to what is classically seen in AIP, her pancreatitis was not steroid responsive.

At this point, a review of the literature was undertaken that included phase III parent and extension clinical trials of belimumab conducted in the past 10 years. We typed search terms “Belimumab” and “Safety” on PubMed and Medline to retrieve studies containing both of the above terms in their title/abstract. We then applied filters for “Type of article" to “Randomized Controlled Trial” and “Publication date” to last 10 years. Out of 25 results, we excluded duplicated results, studies with children subjects and studies in which belimumab was used for the treatment of lupus nephritis, ANCA-vasculitis and rheumatoid arthritis. Studies describing pharmacokinetics and medication interaction of belimumab were also excluded. Studies with a focus on evaluating efficacy and adverse effect profile were selected and a table of 8 studies was formulated. (Table 1 in the "Discussion" section)

The results and conclusions from these studies on the efficacy and safety of belimumab were studied in detail. Following this, the current FDA data of ongoing Phase IV clinical trials of belimumab was analyzed. It was found that there have, indeed, been reports of acute pancreatitis from belimumab. These cases were especially reported by females in the age bracket 40-49 who had been taking the drug for 1-2 years.

Our patient's hospital course had been complicated by respiratory failure which required intubation, development of a pseudocyst, vocal cord paralysis necessitating percutaneous endoscopic gastrostomy (PEG) tube. A month after discharge, the patient was admitted again with unresolved pancreatitis and enlarging pancreatic pseudocyst. The patient underwent Interventional radiology (IR)-guided drainage of the pseudocyst. The patient ultimately underwent exploratory laparotomy with pancreatic necrosectomy. Even after that, the patient had redevelopment of intra-abdominal pancreatic pseudocysts necessitating left upper quadrant IR drain placement. At the time of the drafting of this case report, the patient was still in the hospital receiving postoperative care.

## Discussion

Belimumab is a fully human recombinant IgG monoclonal antibody that targets and inhibits soluble B-lymphocyte stimulator (BLyS), an essential growth factor for B-cell maturation and activation. Belimumab binds to BLyS and prevents it from attaching to its receptor on the B-cell, allowing for apoptosis and downregulation of circulating B-cell clones [4]. Belimumab can be administered intravenously or subcutaneously.

In the pathogenesis of SLE, many B cells are unable to distinguish themselves from foreign antigens and produce antibodies that target self-antigens causing an overactive autoimmune response [4,5]. Studies have shown that SLE patients have significantly greater levels of BLyS than healthy controls. BlyS levels also have been found to correlate with SLE disease activity [6,7]. These studies provide evidence suggesting that BLyS is a legitimate treatment target in SLE.

Table 1 lists the efficacy and safety data from the three landmark phase III clinical trials of belimumab, i.e, BLISS 52, BLISS 76 and BLISS SC along with data from their extension trials and subgroup analyses. 

**Table 1:** Table of studies included in literature review SLE: systemic lupus erythematosus; AE: adverse event; RCT: randomized controlled trial

	Study	Year of publication	Type	(n)	Objective	Belimumab effective in study population?	Safety profile
1	Efficacy and safety of belimumab in patients with active systemic lupus erythematosus: a randomized, placebo-controlled, phase 3 trial (BLISS-52) [8]	2011	Multicenter RCT	867	Comparing SLE responder index (SRI) in belimumab (1 mg/kg and 10 mg/kg) vs placebo group over 52 weeks.	Yes.	Rates of adverse events comparable in all groups (8%,4%,6%)
2	A Phase III, Randomized, Placebo-Controlled Study of Belimumab, a Monoclonal Antibody that Inhibits B Lymphocyte Stimulator, in patients With Systemic Lupus Erythematosus (BLISS-76) [9]	2011	Multicenter RCT	819	Extension of BLISS-52. Second phase III clinical trial of belimumab for 76 weeks.	Yes with 10 mg/kg belimumab, but not with 1 mg/kg belimumab.	Depression and cancer were reported more frequently with belimumab (6-7%) than with placebo (4%). 6 patients in the belimumab group developed malignancies.
3	A Randomized, Double-Blind, Placebo-Controlled, 52-Week Study of the Efficacy and Safety of Belimumab Administered Subcutaneously Plus Standard Care to Patients with Systemic Lupus Erythematosus (SLE) (BLISS-SC). [10]	2015	Phase III RCT	839	Similar to BLISS-52 except mode of administration of belimumab was subcutaneous.	Yes.	Rates of adverse events comparable in both groups. (10.8% in the belimumab group vs 15.7% in the placebo group). Most commonly reported adverse events were infections and infestations and less common were depression and suicidal ideation. Rate of AEs were highest in the highest body weight quartile.
4	Efficacy and Safety of Subcutaneous Belimumab in Anti-Double-Stranded DNA-Positive, Hypocomplementemia Patients with Systemic Lupus Erythematosus. [11]	2018	Subgroup analysis of BLISS-SC	356	Studying parameters in a subset of patients with SLE and hypocomplementemia defined as C3 <90 mg/dl and/or C4 <10 mg/dl, and anti-double-stranded DNA positive defined as ≥30 IU/ml at baseline.	Yes.	No new safety issues identified.
5	Efficacy and safety of intravenous belimumab in Japanese patients with systemic lupus erythematosus: A subgroup analysis of a phase 3 randomized placebo-controlled trial. [12]	2019	Subgroup analysis of BLISS-52 and 76	60	Studying parameters in the Japanese subset of the study population.	Yes.	No new safety issues identified.
6	Long-Term Safety and Efficacy of Belimumab in Patients with Systemic Lupus Erythematosus: A Continuation of a Seventy-Six-Week Phase III Parent Study in the United States. [13]	2018	Continuation study of BLISS-76	268	Assessing long-term safety and efficacy of IV belimumab in patients followed over 8 years.	Yes.	Primary outcome i.e., frequency of adverse events and organ damage remained stable or declined through year 7. A total of 11 deaths occurred, of which, one was due to cardiogenic shock, possibly related to belimumab.
7	A 6-month open-label extension study of the safety and efficacy of subcutaneous belimumab in patients with systemic lupus erythematosus. [14]	2018	24-week, open-label extension following BLISS-SC.	622	Evaluating the safety, tolerability and efficacy of SC belimumab in patients with SLE beyond 1 year.	Yes.	The proportion of patients experiencing more than one adverse event or serious adverse events were similar in both groups i.e., the placebo-to belimumab group and the belimumab group.
8	Long-term safety profile of belimumab plus standard therapy in patients with systemic lupus erythematosus. [15]	First published in 2012	4-year extension study of BLISS-52	296	Evaluating efficacy and safety profile of IV belimumab over 4 years.	Yes.	Incidence rates of AEs, severe/serious AEs, infusion reactions, infections, malignancies were stable or declined during 4-year belimumab exposure

Ongoing monitoring of the long-term safety of belimumab is imperative. The OBSErve study was an observational cohort study that allowed 92 rheumatologists to report clinical outcomes and overall response patterns in patients receiving belimumab in real-world clinical settings. Out of the 501 ITT (intention-to-treat) population, data was available for 277 patients, who received > 8 infusions of belimumab plus SoC. Among the ITT, a ≥50% improvement in overall clinical response between baseline and month 6 was reported for 48.7% of patients and continued improvement was seen at all subsequent 6-month intervals relative to the previous time point [16].

A case report describes a 40-year-old female with SLE who developed progressive multifocal leukoencephalopathy (PML) after 10 infusions of belimumab [17]. The website “eHealthMe.com” tracks the incidence of adverse events from drugs by allowing everyone to run phase IV clinical trials. According to the website, 14100 people reported side effects when taking belimumab. Among them, 29 people (0.21%) reported acute pancreatitis. In 2020, 20 cases of acute pancreatitis were reported [18]. Time on belimumab when patients had acute pancreatitis was in 1-2 years for 52% of the patients and 1-6 months for 40% of the patients. 96% of the patients were females. The age group at which it is most reported was 40-49 years.

We did not find any studies to support an association between the use of belimumab and acute pancreatitis. It can be theorized that since belimumab decreases the ability of B-cells to distinguish self and foreign antigens, it causes self-destruction of the pancreatic cells that may manifest clinically as acute pancreatitis in some patients. Our patient had acute pancreatitis that kept recurring despite medical or surgical treatment and was only temporarily responsive to steroids. The only event that preceded the development of acute pancreatitis in her case was the addition of belimumab. The recurring nature of her pancreatitis and the temporal sequence of events is what made us dive into literature for a possible association between the two events.

## Conclusions

Our case report and the review of literature reinforces the need for more phase IV trials to fully characterize the incidence, pathophysiology and overall nature of a possible side effect of acute pancreatitis from the use of belimumab.
